# Modeling Parkinson’s Disease Using Induced Pluripotent Stem Cells

**DOI:** 10.1007/s11910-012-0270-y

**Published:** 2012-04-28

**Authors:** Blake Byers, Hsiao-lu Lee, Renee Reijo Pera

**Affiliations:** 1 Institute for Stem Cell Biology and Regenerative Medicine, Stanford University, Palo Alto, CA USA; 2 Department of Bioengineering, Stanford University, Palo Alto, CA 94305 USA; 3 Google Ventures, 1489 Charleston Road, Mountain View, CA 94043 USA; 4 Department of Obstetrics and Gynecology, Stanford University, Stanford, CA USA

**Keywords:** Induced pluripotent stem cells, Parkinson’s disease, Disease modeling, Neurodegenerative disease, Development, Phenotype identification, α-Synuclein, Ubiquitin, Lewy bodies, Protein aggregation

## Abstract

Our understanding of the underlying molecular mechanism of Parkinson’s disease (PD) is hampered by a lack of access to affected human dopaminergic (DA) neurons on which to base experimental research. Fortunately, the recent development of a PD disease model using induced pluripotent stem cells (iPSCs) provides access to cell types that were previously unobtainable in sufficient quantity or quality, and presents exciting promises for the elucidation of PD etiology and the development of potential therapeutics. To more effectively model PD, we generated two patient-derived iPSC lines: a line carrying a homozygous *p.G2019S* mutation in the *leucine-rich repeat kinase 2* (*LRRK2*) gene and another carrying a full gene triplication of the α-synuclein encoding gene, *SNCA*. We demonstrated that these PD-linked pluripotent lines were able to differentiate into DA neurons and that these neurons exhibited increased expression of key oxidative stress response genes and α-synuclein protein. Moreover, when compared to wild-type DA neurons, *LRRK2-G2019S* iPSC-derived DA neurons were more sensitive to caspase-3 activation caused by exposure to hydrogen peroxide, MG-132, and 6-hydroxydopamine. In addition, *SNCA*-triplication iPSC-derived DA neurons formed early ubiquitin-positive puncta and were more sensitive to peak toxicity from hydrogen peroxide-induced stress. These aforementioned findings suggest that *LRRK2-G2019S* and *SNCA*-triplication iPSC-derived DA neurons exhibit early phenotypes linked to PD. Given the high penetrance of the homozygous *LRRK2* mutation, the expression of wild-type α-synuclein protein in the *SNCA*-triplication line, and the clinical resemblance of patients afflicted with these familial disorders to sporadic PD patients, these iPSC-derived neurons may be unique and valuable models for disease diagnostics and development of novel pharmacological agents for alleviation of relevant disease phenotypes.

## Introduction

Neurodegenerative diseases are among the most intractable of human disorders. Over 100 years after Alois Alzheimer gave his first lecture on a patient with “a peculiar disorder of the cerebral cortex,” and almost 200 years since James Parkinson published his first description on the “shaking palsy,” we continue to search for the mechanisms that underlie the neurodegeneration in these eponymous diseases.

Pathologically, the neurodegenerative disorders include Parkinson’s disease (PD), Alzheimer’s disease (AD), Huntington’s disease, amyotrophic lateral sclerosis, frontotemporal dementia (FTD), and prion disease and are characterized by protein aggregation, inclusion body formation, and eventual significant neuronal loss. Our understanding of neurodegenerative disease pathogenesis has been spurred by the convergence of two disciplines: the molecular characterization of aggregating proteins in the diseased brain, and the identification of familial forms of AD, PD, and FTD.

PD is a debilitating neurodegenerative disorder characterized by a loss of neurons in the central nervous system (CNS), particularly the dopaminergic neurons of the substantia nigra pars compacta [1–6], but also in the locus coeruleus, nucleus basalis of Meynert, and the dorsal nucleus of the vagus nerve [7]. The majority of PD cases result from unknown etiology, with the loss of dopaminergic neurons of the substantia nigra pars compacta disrupting the basal ganglia network of the midbrain, striatum, and cortex. The disruption of the basal ganglia pathway leads to reduced thalamus activity, which in turn fails to adequately control cortical function through glutamatergic activation. The most noticeable and severe consequence of this signal disruption is a gradual decrease of motor cortex activity, especially in the primary motor cortex and the supplementary motor area. This gives rise to the cardinal PD clinical features of bradykinesia, rigidity, and tremor, while loss of dopaminergic neurons in the olfactory bulb can lead to impairment of the sense of smell [1–6, 8–11].

In PD, protein aggregates known as Lewy bodies and Lewy neurites form in the neuronal soma and neuritic projections, respectively. Since the identification of these lesions in the early 1920s and their subsequent characterization by electron microscopy as structures comprised of abnormal filaments in the 1960s, the presence of these protein aggregates was considered integral to the observed progressive CNS degeneration. However, the molecular mechanisms underlying the initiation and progression of PD remain largely elusive. Instrumental in our disease model conception has been the identification of patients with genetic variants that lead to autosomal-recessive or -dominant forms of the disease. Mutations in *GBA*, *LRRK2*, *PARK2*, *PARK7* (*DJ-1*), *PINK1*, *SNCA*, or *UCHL1* can lead to monogenic forms of PD or increased PD susceptibility, suggesting important roles for these proteins in the pathogenesis of the disease [12–17]. Of the distinct PD-related genetic alterations discovered, 500 relate to the five most prominently represented genes: *SNCA*, *LRRK2*, *PARK2*, *PINK1*, and *PARK7*, with genetic alterations in *SNCA* and *LRRK2* leading to autosomal-dominant forms of the disease [12]. Notably, the genes identified in familial forms of PD, FTD, and AD are linked to the proteins that aggregate in both inherited and sporadic forms of those diseases.

The first such mutation identified was a *p.Ala53Thr* mutation in the *SNCA* gene, which encodes α-synuclein, while subsequent studies have found that other *SNCA* mutants cause other inherited forms of PD. Intriguingly, Lewy bodies were independently found to be principally composed of the α-synuclein protein [18]. The existence of autosomal-dominant *SNCA* mutations in combination with the aggregation of the α-synuclein protein in the diseased brain, suggests a key and perhaps causal role for this protein—a conclusion that may extend to sporadic forms of PD.

The remaining products of PD-related genes do not natively aggregate, but they can be linked in direct and indirect ways to protein degradation pathways: *GBA* encodes the key lysosomal protein β-glucocerebrosidase; *LRRK2* encodes an enzyme called *leucine-rich repeat kinase 2* that has a predicted kinase activity, but is associated with the organization of lysosomes, endosomes, and other membranous organelles; *PARK2* is part of the E3 ubiquitin-ligase complex; *PARK7* protects cells against oxidative stress; *PINK1* is hypothesized to be protective against cell stress by phosphorylating mitochondrial proteins; *UCHL1* is a neural-specific ubiquitin-protein hydrolase. Taken together, the genes affected by familial PD mutations yield an important foundation on which to build our understanding of this neurodegenerative disease.

## α-Synuclein and *LRRK2*

α-Synuclein is a 17-kD protein with two human homologs, β-synuclein and γ-synuclein. Robustly expressed throughout the CNS and peripheral nervous system (PNS), α-synuclein has long been considered to be a natively unstructured protein that is capable of taking on partially folded amyloidogenic α-helical or β-sheet conformations [19]. However, more recent studies challenge this prevailing paradigm, by suggesting that endogenous α-synuclein in non-denaturing conditions may form helically folded soluble tetramers that resist aggregation [20••].

Although the exact function of α-synuclein remains unknown, the protein has been found in presynaptic vesicles, thus leading to its original nomenclature from the Torpedo ray. Further, α-synuclein has been shown to bind to lipids, potentially via its imperfect 11 amino-acid repeats, and associate with phospholipase D2 [21–23]. Taken together, these results implicate α-synuclein in neurotransmitter release, vesicle turnover, channel localization, and dose-dependent dynamic dopamine release [24]. Recently, Chandra et al. [25] and Burré et al. [26] demonstrated that α-synuclein is required for presynaptic vesicle chaperoning through SNARE-complex assembly by directly binding to VAMP2. These findings further support a neural-specific role for α-synuclein at the level of presynaptic vesicles.

α-Synuclein knockout animals appear to be relatively unaffected—with conflicting literature reports suggesting neuroprotective effects, mitochondrial degeneration resistance, and neurotransmitter deficits [27, 28]. However, the importance of α-synuclein should not be underestimated; the presence and similarity of β-synuclein and γ-synuclein in these knockout animals may compensate for the deficiency of α-synuclein, masking critical processes that it is involved in. Recent studies on complete synuclein-knockout animals showed age-dependent neurological impairments with premature death [29].

While *SNCA* whole-locus multiplications (duplication or triplications) are perhaps the most relevant to sporadic patients, as they encode a qualitatively wild-type protein rather than a missense form, *LRRK2* mutations are, by far, the most common inherited form of PD. Studies in animal and cellular models have shown that certain mutations in the *LRRK2* gene can increase its kinase activity, which contributes to neurotoxicity, possibly via the oxidative stress pathway [30–33]. Moreover, cells expressing mutant *LRRK2* have been shown to be more vulnerable to peroxide-induced cell death than wild-type *LRRK2* cells [6, 32]. Although the function of *LRRK2* is currently not fully understood, there is evidence that the reduced expression of *LRRK2* mRNA limits neural progenitor cell differentiation toward dopaminergic neurons and may enhance cell death [34]. These findings suggest that *LRRK2* plays a pivotal role in neuronal survival, and that the loss of function in this gene might also be pathogenic for PD [35].

## Models of Parkinson’s Disease

To understand the development and progression of a human disease, an effective model that combines the genetic underpinnings with a similar, if not identical, phenotypic output is required. The appreciable gaps in our understanding of the mechanisms of PD initiation and progression, along with other sporadic and genetic neurodegenerative diseases, can be traced to the current lack of accurate models of these complex human diseases. The standard process for modeling human disease with genetic origins has been to overexpress the human gene of interest in model organisms (from yeast, to drosophila, to mouse, to non-human primate) and then observe the cellular and protein response. Perturbations that appear phenotypic are used as the fitness function for subsequent drug screening or therapeutic target selection. These studies have provided some important advances for a large spectrum of human disease, and have been instrumental in our basic understanding of neurodegenerative diseases [33, 36–39].

However, the use of these transgenic models have failed to develop an accurate portrait of the underlying disease mechanisms. This unfortunate result should not be surprising. While overexpression of human genes in model organisms may constitute an accurate representation of the underlying human pathogenesis for some diseases, in complex neurodegenerative diseases that involve endogenously expressed human proteins, basic cellular machinery such as transcriptional feedback, and stochastic balance of the proteasomal pathway are bound to play significant roles. These two fundamental cellular processes are greatly compromised in overexpression organism models: transcriptional feedback is impossible when proteins are expressed on a non-native promoter, or even a native promoter in a different location in the genome, where epigenetics and other sequence neighbor effects will undoubtedly play a role in expression. The result is that mechanistic studies of neurodegeneration have been thwarted by an inability to study the cellular processes in susceptible human neurons that had an endogenous predisposition to the disease.

The lack of studies employing human cells to investigate human neurodegenerative disease is not due to a lack of interest. PD is challenging to study due to the inaccessibility of affected human midbrain dopaminergic (mDA) neurons and a scarcity of animal models that mimic the key disease features. The transgenic animals and cellular models expressing known PD-associated genes have provided important insights into the pathogenesis of the disease; however, it has been difficult to demonstrate that the implicated mechanisms are also present in neurons from affected individuals.

Previous genetic studies have implicated several potential mechanisms but the pathogenesis of PD remains largely obscure. In most PD patients, Lewy bodies and Lewy neurites form in both CNS and autonomic PNS neurons. These large intracellular proteinaceous inclusions are rich in α-synuclein and ubiquitin, an observation that suggests a role for α-synuclein and the proteasome in the molecular development of sporadic and inherited PD [40]. However, not all patients with PD develop Lewy bodies. Although mutations in *LRRK2* are commonly associated with PD and Lewy body pathology, some of these patients do not exhibit a Lewy phenotype. Therefore, it is thought that protein aggregration may actually be a disease modifier rather than direct cause [41]. Methods to identify patients with a well-defined aggregation phenotype may be useful in categorizing disease, as well as in correctly targeting disease-modifying interventions to an appropriate class of patients.

As discussed, autosomal-dominant forms of PD have been documented in families with *SNCA* missense mutations or gene duplication/triplications. α-Synuclein protein aggregation and markers of cellular stress are also seen in other neurodegenerative disorders, including multiple system atrophy, Bradbury-Eggleston syndrome, and dementia with Lewy bodies.

The selective degeneration of neural subtypes in each disease has since been linked to the resulting cornucopia of observed clinical manifestations. However, recent evidence suggests that the true culprit of cellular toxicity may be the soluble cytoplasmic oligomeric α-synuclein protein, whereas the large insoluble protein aggregates may in fact represent a cellular defense mechanism in which the cell sequesters cytotoxic-soluble oligomeric proteins into insoluble inclusion bodies [42].

Attempting to replicate the cardinal phenotypic features of PD, researchers have made use of animal models of dopaminergic neurotoxicity, and transgenic models of the familial PD-causing genes. In drosophila, overexpression of SNCA leads to age-dependent dopaminergic neuron degeneration. Also, transgenic mice overexpressing mouse and human SNCA exhibit pathological phenotypes—inclusion body formation in some CNS cells, slight impairment in motor control, and loss of dopaminergic neuron terminals in the basal ganglia. These results from transgenic models support a causal role for α-synuclein in the development of PD [43–45]. Furthermore, the experiments using *LRRK2-G2019S* transgenic mouse models demonstrated age-dependent and levodopa-responsive slowness of movement associated with diminished dopamine release and axonal pathology [46].

Taking together this body of evidence and the known inherited forms of PD, one can construct a basic PD model: α-synuclein begins to aggregate, through some innate mechanism, or induced by other factors; the cell recognizes the buildup of cytosolic α-synuclein levels and attempts to degrade the proteins by ubiquitination, and subsequent shuttling to a proteasome for degradation. In the diseased brain, any one of the following processes could be disturbed: the innate dynamic aggregation of α-synuclein, the regulation of α-synuclein expression level, ubiquitination of α-synuclein, and degradation of α-synuclein degradation. Interestingly, Lewy bodies generally contain high concentrations of ubiquitin, suggesting unfruitful attempts by the cellular proteasomal pathway to degrade α-synuclein despite substantial poly-ubiquitination.

In sum, while it has been shown that overexpression of α-synuclein transgenes leads to protein aggregation in normal cells, the study of native processes leading to aggregation in affected individuals has been hindered by the inaccessibility of human neurons in vivo, the limitations inherent in studying postmortem samples from PD patients, and the inability to accurately recapitulate human disease in transgenic models [47–49].

## Induced Pluripotent Stem Cells

The development of human induced pluripotent stem cells (iPSCs) in 2007 by Takahashi et al. [50] and Yu et al. [51] has presented a novel approach to study human diseases. Where researchers were previously restricted to studying human proteins in virally induced overexpression animal models, or, at best, a zinc-finger cleaved recombination on a native promoter, we are now able to generate stable pluripotent lines from patients with inherited forms of disease, with their endogenous genome and its transcriptional feedbacks largely intact.

To explore the potential use of iPSC-derived neurons in detecting protein accumulation and aggregation phenotypes, we selected two PD-causing genotypes: the homozygous *G2019S* point mutation in the *LRRK2* gene, which causes autosomal-dominant PD, and the heterozygous triplication of the *SNCA* locus (total: four functional gene copies), which also causes autosomal-dominant PD. In keeping with a dominant mode of inheritance, in the vast majority of LRRK2-PD patients a single heterozygous *G2019S* mutation is present; however, rare patients with homozygous *G2019S* mutation have also been reported (mostly due to parental consanguinity), resulting in a similar phenotype.

*LRRK2-G2019S* had previously been identified in 0.6–1.6% and 2–8% of sporadic and familial PD, respectively [52–54]. The penetrance of this particular mutation is age-dependent, increasing from 17% penetrance at age 50–85% at age 70 years [55]. Clinical symptoms associated with PD in individuals carrying the *LRRK2-G2019S* mutation are mostly indistinguishable from those affected by late-onset idiopathic PD with pathologies affecting both motor and non-motor bodily functions [56].

α-Synuclein expression level is known to influence disease progression with triplications causing earlier onset and more rapid progression than duplications [17, 49, 57, 58]. Due to this pronounced phenotype in patients, *SNCA* triplication genotype was selected to provide the highest probability of detecting disease-related phenotypes that may be useful in categorizing disease characteristics in patient-specific iPSC-derived neurons.

Although derivation of iPSC lines from idiopathic PD patients has been reported, it is still not known whether these PD iPSCs can give rise to mDA neurons that exhibit native phenotypes related to PD in vitro [59, 60]. As PD is likely to result from a combination of genetic and environmental factors, it is not clear to what extent sporadic PD lines will demonstrate phenotypes that are clearly discernible; thus, proof-of-principle demonstrations that iPSC lines recapitulate key phenotypes of PD may be most effectively achieved using cells from patients with monogenic mutations. Recent work by Israel et al. [61••] has demonstrated the utility of iPSC modeling for AD, suggesting iPSC modeling may be broadly applicable to neurodegenerative diseases. Generation of PD phenotypes from iPSC-derived neurons would be useful for studies aimed at understanding disease mechanism, prevention, and treatment.

## Conclusions

iPSCs offer a new platform for studying human diseases in the most endogenous form possible when patient primary cell lines are unavailable [62•, 63•]. Further, differentiation and maturation of the IPSCs enable the study of disease phenotypes during development and aging of the cell type of interest. Using this approach, we have demonstrated a causal role of SNCA-triplication and LRRK2-G2019S in developing PD-related phenotypes. Broadly speaking, iPSC models may be readily extended to other neurodegenerative diseases with genomic mutations underpinnings, and is not just limited to PD [61••, 64, 65].
